# Crystal structure of bis­[μ-1,2-bis­(di­phenyl­phosphan­yl)ethane-κ^2^ *P*:*P*′]bis[(*N*,*N*′-di­ethyl­thio­urea-κ*S*)iodidocopper(I)]

**DOI:** 10.1107/S2056989015014176

**Published:** 2015-08-06

**Authors:** Ladawan Khongsichan, Arunpatcha Nimthong-Roldán, Chaveng Pakawatchai, Sumpun Wongnawa

**Affiliations:** a Department of Chemistry, Faculty of Science, Prince of Songkla University, Hat Yai, Songkhla 90112, Thailand; b Department of Chemistry, Youngstown State University, 1 University Plaza, 44555 Youngstown, OH, USA

**Keywords:** crystal structure, copper(I) complex, *N*,*N*′-di­ethyl­thio­urea, N—H⋯I hydrogen bonding

## Abstract

The binuclear title complex, [Cu_2_I_2_(C_26_H_24_P_2_)_2_(C_5_H_12_N_2_S)_2_], lies about an inversion centre. The Cu^I^ atom displays a distorted tetra­hedral coordination geometry defined by one S atom of an *N*,*N*′-di­ethyl­thio­urea ligand, two P atoms derived from two bridging 1,2-bis­(di­phenyl­phosphan­yl)ethane (dppe) ligands and one iodide ion. The dppe ligand bridges two symmetry-related Cu^I^ ions, forming a 10-membered Cu_2_P_4_C_4_ ring. An intra­molecular N—H⋯I hydrogen bond is noted. In the crystal, N—H⋯I hydrogen bonds link complex mol­ecules into layers parallel to (-101).

## Related literature   

For background to the coordination chemistry of copper(I) halides and pseudohalides, see: Dennehy *et al.* (2011 ▸); Oshio *et al.* (1996 ▸); Seward *et al.* (2003 ▸). For their potential applications, see: Corey *et al.* (1987 ▸); Dias *et al.* (2006 ▸). For relevant examples of discrete complexes, see: Dennehy *et al.* (2009 ▸).
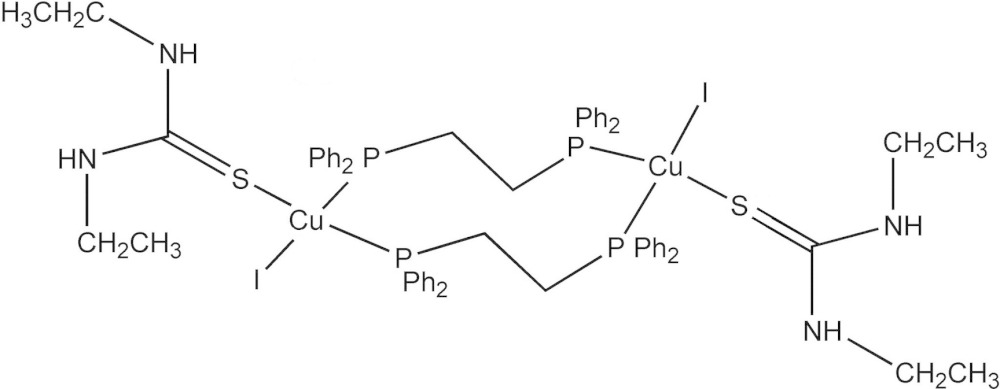


## Experimental   

### Crystal data  

[Cu_2_I_2_(C_26_H_24_P_2_)_2_(C_5_H_12_N_2_S)_2_]
*M* *_r_* = 1442.11
Monoclinic, 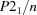

*a* = 12.2150 (8) Å
*b* = 15.1836 (9) Å
*c* = 17.1801 (10) Å
β = 96.414 (2)°
*V* = 3166.4 (3) Å^3^
*Z* = 2
Cu *K*α radiation
μ = 10.37 mm^−1^
*T* = 100 K
0.16 × 0.15 × 0.08 mm

### Data collection  

Bruker Prospector CCD diffractometer
Absorption correction: multi-scan (*SADABS*; Bruker, 2013 ▸) *T*_min_ = 0.433, *T*_max_ = 0.753
23236 measured reflections
5564 independent reflections
5556 reflections with *I* > 2σ(*I*)
*R*_int_ = 0.028

### Refinement  

*R*[*F* ^2^ > 2σ(*F* ^2^)] = 0.025
*wR*(*F* ^2^) = 0.063
*S* = 1.13
5564 reflections
345 parameters
H-atom parameters constrained
Δρ_max_ = 0.98 e Å^−3^
Δρ_min_ = −0.92 e Å^−3^

### 

Data collection: *APEX2* (Bruker, 2013 ▸); cell refinement: *SAINT* (Bruker, 2013 ▸); data reduction: *SAINT*; program(s) used to solve structure: *SHELXS97* (Sheldrick, 2008 ▸); program(s) used to refine structure: *SHELXL2015* (Sheldrick, 2015 ▸) and *SHELXLE* (Hübschle *et al.*, 2011 ▸); molecular graphics: *Mercury* (Macrae *et al.*, 2008 ▸); software used to prepare material for publication: *publCIF* (Westrip, 2010 ▸).

## Supplementary Material

Crystal structure: contains datablock(s) I. DOI: 10.1107/S2056989015014176/tk5371sup1.cif

Structure factors: contains datablock(s) I. DOI: 10.1107/S2056989015014176/tk5371Isup2.hkl

**Fig. 1.**
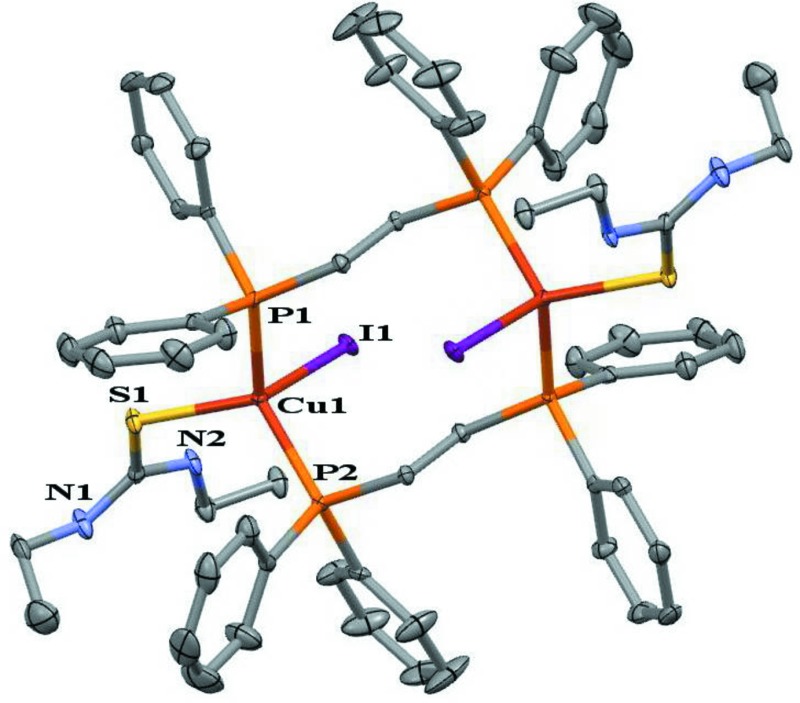
The structure of title complex with displacement ellipsoids drawn at the 50% propbability level. All H atoms are omitted for clarity.

. DOI: 10.1107/S2056989015014176/tk5371fig1.tif
The structure of title complex with displacement ellipsoids drawn at the 50% propbability level. All H atoms are omitted for clarity.

**Fig. 2.**
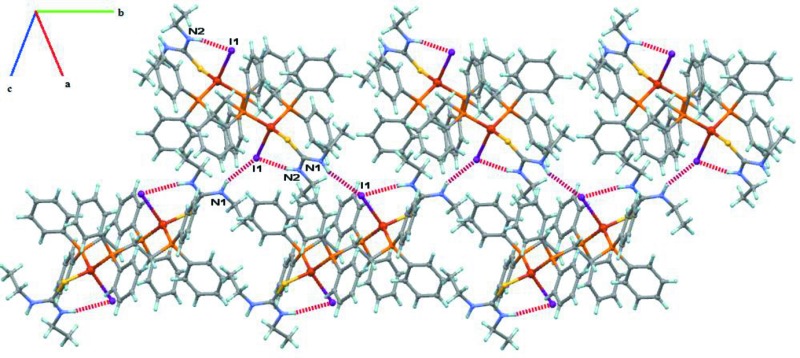
Part of the crystal structure showing intra/inter-molecular N—H···I hydrogen bonds forming a layers as dashed lines.

. DOI: 10.1107/S2056989015014176/tk5371fig2.tif
Part of the crystal structure showing intra/inter-mol­ecular N—H⋯I hydrogen bonds forming a layers as dashed lines.

CCDC reference: 1415379

Additional supporting information:  crystallographic information; 3D view; checkCIF report

## Figures and Tables

**Table 1:** Hydrogen-bond geometry (, )

*D*H*A*	*D*H	H*A*	*D* *A*	*D*H*A*
N1H1I1^i^	0.88	2.80	3.622(2)	156
N2H2I1	0.88	2.70	3.5517(19)	162

Symmetry code: (i) 
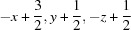
.

## supplementary crystallographic information

### S0.1. Synthesis and crystallization

*N*,*N*'-Di­ethyl­thio­urea (0.07 g, 0.5 mmol) was dissolved in 30 cm^3^ of aceto­nitrile in a round flask equipped with reflux condenser and magnetic stirrer at 333 K and then CuI (0.1 g, 0.5 mmol) was added. The mixture was stirred for 2 h. 1,2-bis­(di­phenyl­phosphanyl)ethane (0.2 g, 0.5 mmol) was added and the reaction mixture was heated under reflux for 5 h where upon the precipitate gradually disappeared. The resulting clear solution was filtered and left to evaporate at room temperature. The colorless crystals, which deposited after standing for several days were filtered off and washed with acetone and dried *in vacuo* (M. pt = 557 K). Elemental analysis, calculated for [Cu_2_I_2_(C_26_H_24_P_2_)_2_(C_5_H_12_N_2_S)_2_]: C, 51.59; H, 4.97; N, 3.88; S, 4.44%, found: C, 55.69; H, 5.22; N, 3.62; S, 4.61%.

### S0.2. Refinement

The (-1 8 3) reflection was affected by the beam-stop and was omitted from the final cycles of refinement. H atoms bonded to C and N atoms were included in their calculated positions and were refined using a riding model using bond lengths of 0.95–0.99 Å and *U*_iso_(H) = 1.2–1.5*U*_eq_(C), and N—H = 0.88 Å (NH) and *U*_iso_(H) = 1.2*U*_eq_(N). The (-1 8 3) reflection was omitted owing to poor agreement.

### S1. Comment

Coordination complexes of copper(I) halides or pseudo-halides with mixed P and S donor ligands have been of inter­est in coordination chemistry (Dennehy *et al*., 2011; Oshio *et al.*, 1996; Sewead *et al.*, 2003) due to their applications such as magnetism (Oshio *et al.*, 1996) and biological or medicinal activities (Corey *et al.*, 1987; Dias *et al.*, 2006). In this work, a mixed ligand complex of copper(I) iodide with 1,2-bis­(di­phenyl­phosphanyl)ethane (dppe) and *N*,*N*'-di­ethyl­thio­urea (detu) is reported. The binuclear copper(I) complex lies across an inversion center. The *µ*_2_-dppe bridges between Cu^I^ centers leads to a 10-membered Cu_2_P_4_C_4_ rhomboid, see Fig. 1. The Cu1—P1 and Cu1—P2 bond lengths are 2.2681 (6) and 2.2813 (6)Å, respectively. These values are slightly shorter than the equivalent distances found in [Cu(tsac)(PPh_3_)_2_], [Cu_4_(tsac)_4_(PPh_3_)_3_], [Cu_2_(tsac)_2_(dppm)_2_] and [Cu_4_(tsac)_4_(dppm)_2_], which are in the range 2.2799 (5) and 2.3119 (5) Å (Dennehy *et al.*, 2009). There is an intra­molecular N2—H2···I1 hydrogen bond. In the crystal, inter­molecular N1—H1···I1 hydrogen bonds link complex molecules into a two-dimensional supra­molecular network parallel to (-101) (Fig. 2. and Table 1)

### Crystal data

**Table:**

[Cu_2_I_2_(C_26_H_24_P_2_)_2_(C_5_H_12_N_2_S)_2_]	*F*(000) = 1456
*M**_r_* = 1442.11	*D*_x_ = 1.513 Mg m^−^^3^
Monoclinic, *P*2_1_/*n*	Cu *K*α radiation, λ = 1.54178 Å
*a* = 12.2150 (8) Å	Cell parameters from 9846 reflections
*b* = 15.1836 (9) Å	θ = 3.9–66.7°
*c* = 17.1801 (10) Å	µ = 10.37 mm^−^^1^
β = 96.414 (2)°	*T* = 100 K
*V* = 3166.4 (3) Å^3^	Block, colourless
*Z* = 2	0.16 × 0.15 × 0.08 mm

### Data collection

**Table:**

Bruker Prospector CCD diffractometer	5564 independent reflections
Radiation source: I-mu-S microsource X-ray tube	5556 reflections with *I* > 2σ(*I*)
Laterally graded multilayer (Goebel) mirror monochromator	*R*_int_ = 0.028
ω and phi scans	θ_max_ = 67.0°, θ_min_ = 3.9°
Absorption correction: multi-scan (*SADABS*; Bruker, 2013)	*h* = −14→12
*T*_min_ = 0.433, *T*_max_ = 0.753	*k* = −18→17
23236 measured reflections	*l* = −20→20

### Refinement

**Table:**

Refinement on *F*^2^	Primary atom site location: structure-invariant direct methods
Least-squares matrix: full	Secondary atom site location: difference Fourier map
*R*[*F*^2^ > 2σ(*F*^2^)] = 0.025	Hydrogen site location: inferred from neighbouring sites
*wR*(*F*^2^) = 0.063	H-atom parameters constrained
*S* = 1.13	*w* = 1/[σ^2^(*F*_o_^2^) + (0.0315*P*)^2^ + 3.4263*P*] where *P* = (*F*_o_^2^ + 2*F*_c_^2^)/3
5564 reflections	(Δ/σ)_max_ = 0.003
345 parameters	Δρ_max_ = 0.98 e Å^−^^3^
0 restraints	Δρ_min_ = −0.92 e Å^−^^3^

### Special details

**Table:**

Geometry. All e.s.d.'s (except the e.s.d. in the dihedral angle between two l.s. planes) are estimated using the full covariance matrix. The cell e.s.d.'s are taken into account individually in the estimation of e.s.d.'s in distances, angles and torsion angles; correlations between e.s.d.'s in cell parameters are only used when they are defined by crystal symmetry. An approximate (isotropic) treatment of cell e.s.d.'s is used for estimating e.s.d.'s involving l.s. planes.

### Fractional atomic coordinates and isotropic or equivalent isotropic displacement parameters (Å^2^)

**Table:**

	*x*	*y*	*z*	*U*_iso_*/*U*_eq_	
I1	0.71658 (2)	0.56133 (2)	0.16309 (2)	0.01039 (6)	
Cu1	0.51704 (2)	0.64264 (2)	0.12681 (2)	0.00798 (8)	
S1	0.48147 (4)	0.74104 (3)	0.22620 (3)	0.01238 (12)	
P1	0.39142 (4)	0.53264 (3)	0.12684 (3)	0.00711 (11)	
P2	0.52134 (4)	0.70642 (3)	0.00696 (3)	0.00768 (11)	
N1	0.58767 (16)	0.88958 (13)	0.27366 (12)	0.0163 (4)	
H1	0.6493	0.9171	0.2902	0.020*	
N2	0.69626 (15)	0.77334 (13)	0.24764 (11)	0.0122 (4)	
H2	0.7019	0.7167	0.2380	0.015*	
C1	0.59546 (18)	0.80593 (15)	0.25058 (12)	0.0109 (4)	
C2	0.4851 (2)	0.93892 (15)	0.27369 (17)	0.0198 (5)	
H2A	0.4929	0.9817	0.3174	0.024*	
H2B	0.4250	0.8977	0.2827	0.024*	
C3	0.4547 (3)	0.9875 (2)	0.19789 (19)	0.0348 (7)	
H3A	0.5135	1.0289	0.1891	0.052*	
H3B	0.3859	1.0199	0.2007	0.052*	
H3C	0.4448	0.9452	0.1546	0.052*	
C4	0.79787 (18)	0.82441 (16)	0.25912 (14)	0.0149 (5)	
H4A	0.8076	0.8487	0.3129	0.018*	
H4B	0.7936	0.8743	0.2218	0.018*	
C5	0.89514 (18)	0.76646 (16)	0.24646 (14)	0.0162 (5)	
H5A	0.8813	0.7370	0.1956	0.024*	
H5B	0.9055	0.7222	0.2881	0.024*	
H5C	0.9617	0.8027	0.2475	0.024*	
C11	0.38548 (18)	0.47611 (14)	0.22045 (13)	0.0103 (4)	
C12	0.45710 (18)	0.50116 (14)	0.28549 (13)	0.0124 (4)	
H12	0.5106	0.5456	0.2807	0.015*	
C13	0.4500 (2)	0.46061 (16)	0.35789 (13)	0.0161 (5)	
H13	0.4989	0.4777	0.4022	0.019*	
C14	0.3723 (2)	0.39590 (16)	0.36541 (14)	0.0196 (5)	
H14	0.3673	0.3692	0.4149	0.024*	
C15	0.3015 (2)	0.36998 (17)	0.30053 (15)	0.0201 (5)	
H15	0.2486	0.3250	0.3055	0.024*	
C16	0.30793 (19)	0.40978 (16)	0.22830 (14)	0.0149 (5)	
H16	0.2594	0.3918	0.1840	0.018*	
C21	0.24971 (18)	0.57205 (14)	0.10553 (14)	0.0109 (4)	
C22	0.1845 (2)	0.55542 (16)	0.03495 (15)	0.0188 (5)	
H22	0.2117	0.5190	−0.0035	0.023*	
C23	0.0792 (2)	0.59235 (18)	0.02086 (16)	0.0240 (5)	
H23	0.0359	0.5819	−0.0277	0.029*	
C24	0.0375 (2)	0.64380 (17)	0.07685 (17)	0.0246 (6)	
H24	−0.0346	0.6678	0.0674	0.029*	
C25	0.1021 (2)	0.66008 (18)	0.14715 (17)	0.0245 (6)	
H25	0.0739	0.6953	0.1860	0.029*	
C26	0.2072 (2)	0.62539 (17)	0.16094 (14)	0.0177 (5)	
H26	0.2510	0.6381	0.2088	0.021*	
C27	0.39162 (19)	0.44094 (13)	0.05650 (13)	0.0098 (4)	
H27A	0.3228	0.4068	0.0578	0.012*	
H27B	0.3910	0.4658	0.0032	0.012*	
C28	0.51050 (17)	0.62207 (14)	−0.07137 (12)	0.0090 (4)	
H28A	0.4406	0.5891	−0.0713	0.011*	
H28B	0.5110	0.6507	−0.1231	0.011*	
C31	0.40910 (19)	0.78228 (15)	−0.02656 (12)	0.0126 (4)	
C32	0.4258 (2)	0.87216 (18)	−0.03567 (19)	0.0302 (6)	
H32	0.4977	0.8963	−0.0252	0.036*	
C33	0.3369 (3)	0.9266 (2)	−0.0601 (2)	0.0432 (8)	
H33	0.3488	0.9880	−0.0658	0.052*	
C34	0.2325 (3)	0.8932 (2)	−0.07599 (18)	0.0343 (7)	
H34	0.1728	0.9310	−0.0933	0.041*	
C35	0.2148 (2)	0.8042 (2)	−0.06672 (16)	0.0279 (6)	
H35	0.1427	0.7805	−0.0779	0.033*	
C36	0.3023 (2)	0.74917 (18)	−0.04106 (15)	0.0205 (5)	
H36	0.2893	0.6883	−0.0333	0.025*	
C41	0.64463 (18)	0.76671 (14)	−0.01297 (13)	0.0114 (4)	
C42	0.7191 (2)	0.79247 (18)	0.04993 (15)	0.0239 (6)	
H42	0.7029	0.7809	0.1018	0.029*	
C43	0.8164 (3)	0.8347 (2)	0.03845 (17)	0.0333 (7)	
H43	0.8658	0.8524	0.0823	0.040*	
C44	0.8419 (2)	0.8513 (2)	−0.03605 (18)	0.0315 (7)	
H44	0.9092	0.8794	−0.0440	0.038*	
C45	0.7686 (3)	0.8267 (3)	−0.09912 (18)	0.0423 (9)	
H45	0.7856	0.8383	−0.1508	0.051*	
C46	0.6708 (3)	0.7853 (2)	−0.08813 (15)	0.0316 (7)	
H46	0.6209	0.7694	−0.1323	0.038*	

### Atomic displacement parameters (Å^2^)

**Table:**

	*U*^11^	*U*^22^	*U*^33^	*U*^12^	*U*^13^	*U*^23^
I1	0.01029 (9)	0.00674 (9)	0.01363 (9)	0.00312 (4)	−0.00089 (6)	−0.00191 (4)
Cu1	0.00902 (16)	0.00693 (16)	0.00820 (15)	−0.00076 (11)	0.00197 (12)	−0.00022 (11)
S1	0.0096 (2)	0.0109 (3)	0.0175 (3)	−0.00149 (19)	0.0049 (2)	−0.0069 (2)
P1	0.0074 (2)	0.0068 (3)	0.0076 (2)	−0.00001 (19)	0.00262 (19)	−0.00070 (19)
P2	0.0097 (3)	0.0057 (2)	0.0078 (2)	0.00084 (19)	0.00168 (19)	0.00046 (19)
N1	0.0105 (9)	0.0122 (10)	0.0262 (11)	−0.0006 (8)	0.0025 (8)	−0.0094 (8)
N2	0.0100 (9)	0.0092 (9)	0.0174 (9)	0.0010 (7)	0.0012 (7)	−0.0073 (7)
C1	0.0121 (10)	0.0117 (11)	0.0096 (10)	0.0003 (8)	0.0036 (8)	−0.0022 (8)
C2	0.0172 (12)	0.0122 (12)	0.0315 (14)	0.0030 (9)	0.0091 (11)	−0.0075 (9)
C3	0.0314 (15)	0.0299 (16)	0.0441 (17)	0.0096 (13)	0.0083 (13)	0.0068 (13)
C4	0.0112 (11)	0.0149 (11)	0.0185 (11)	−0.0007 (9)	0.0012 (9)	−0.0063 (9)
C5	0.0105 (10)	0.0172 (12)	0.0208 (12)	−0.0002 (9)	0.0011 (9)	−0.0089 (9)
C11	0.0117 (10)	0.0088 (10)	0.0112 (10)	0.0041 (8)	0.0052 (8)	0.0014 (8)
C12	0.0144 (10)	0.0090 (10)	0.0146 (11)	0.0020 (8)	0.0051 (9)	−0.0004 (8)
C13	0.0214 (12)	0.0149 (11)	0.0124 (11)	0.0049 (10)	0.0030 (9)	−0.0003 (9)
C14	0.0248 (12)	0.0197 (13)	0.0164 (11)	0.0054 (10)	0.0115 (10)	0.0085 (9)
C15	0.0170 (11)	0.0186 (12)	0.0260 (13)	−0.0024 (10)	0.0088 (10)	0.0076 (10)
C16	0.0106 (10)	0.0166 (12)	0.0181 (11)	−0.0015 (9)	0.0036 (9)	0.0029 (9)
C21	0.0088 (10)	0.0093 (10)	0.0151 (11)	−0.0017 (8)	0.0033 (9)	0.0016 (8)
C22	0.0156 (12)	0.0219 (13)	0.0185 (12)	0.0035 (9)	0.0002 (10)	−0.0044 (9)
C23	0.0166 (12)	0.0239 (14)	0.0297 (14)	0.0046 (11)	−0.0059 (10)	0.0002 (11)
C24	0.0120 (11)	0.0191 (13)	0.0425 (16)	0.0048 (10)	0.0023 (11)	0.0007 (11)
C25	0.0191 (12)	0.0202 (13)	0.0358 (15)	0.0057 (10)	0.0105 (11)	−0.0064 (11)
C26	0.0175 (12)	0.0161 (12)	0.0200 (12)	0.0017 (9)	0.0042 (10)	−0.0048 (9)
C27	0.0112 (11)	0.0082 (11)	0.0101 (10)	0.0002 (8)	0.0017 (8)	−0.0020 (7)
C28	0.0111 (10)	0.0075 (10)	0.0085 (10)	0.0002 (8)	0.0016 (8)	−0.0017 (8)
C31	0.0175 (11)	0.0123 (11)	0.0088 (10)	0.0062 (9)	0.0039 (8)	0.0011 (8)
C32	0.0274 (14)	0.0171 (13)	0.0456 (17)	0.0059 (11)	0.0025 (12)	0.0054 (12)
C33	0.045 (2)	0.0210 (15)	0.064 (2)	0.0149 (14)	0.0092 (17)	0.0142 (15)
C34	0.0319 (15)	0.0399 (18)	0.0315 (15)	0.0273 (14)	0.0049 (12)	0.0107 (13)
C35	0.0188 (13)	0.0409 (17)	0.0238 (13)	0.0122 (12)	0.0018 (10)	−0.0045 (12)
C36	0.0179 (12)	0.0221 (13)	0.0218 (12)	0.0057 (10)	0.0042 (10)	−0.0024 (10)
C41	0.0149 (11)	0.0054 (10)	0.0140 (11)	−0.0001 (8)	0.0027 (9)	0.0020 (8)
C42	0.0278 (14)	0.0278 (14)	0.0154 (12)	−0.0125 (11)	0.0003 (10)	0.0057 (10)
C43	0.0305 (15)	0.0388 (17)	0.0285 (15)	−0.0211 (13)	−0.0067 (12)	0.0108 (13)
C44	0.0245 (14)	0.0330 (16)	0.0371 (16)	−0.0154 (12)	0.0042 (12)	0.0131 (13)
C45	0.0434 (18)	0.063 (2)	0.0220 (14)	−0.0303 (17)	0.0116 (13)	0.0075 (14)
C46	0.0358 (16)	0.0455 (18)	0.0128 (12)	−0.0229 (14)	0.0002 (11)	0.0039 (12)

### Geometric parameters (Å, º)

**Table:**

I1—Cu1	2.7412 (4)	C21—C26	1.394 (3)
Cu1—P1	2.2681 (6)	C21—C22	1.398 (4)
Cu1—P2	2.2813 (6)	C22—C23	1.399 (4)
Cu1—S1	2.3457 (6)	C22—H22	0.9500
S1—C1	1.719 (2)	C23—C24	1.380 (4)
P1—C21	1.829 (2)	C23—H23	0.9500
P1—C11	1.832 (2)	C24—C25	1.389 (4)
P1—C27	1.844 (2)	C24—H24	0.9500
P2—C41	1.827 (2)	C25—C26	1.384 (4)
P2—C31	1.834 (2)	C25—H25	0.9500
P2—C28	1.852 (2)	C26—H26	0.9500
N1—C1	1.337 (3)	C27—C28^i^	1.530 (3)
N1—C2	1.459 (3)	C27—H27A	0.9900
N1—H1	0.8800	C27—H27B	0.9900
N2—C1	1.333 (3)	C28—C27^i^	1.530 (3)
N2—C4	1.458 (3)	C28—H28A	0.9900
N2—H2	0.8800	C28—H28B	0.9900
C2—C3	1.507 (4)	C31—C32	1.391 (4)
C2—H2A	0.9900	C31—C36	1.394 (4)
C2—H2B	0.9900	C32—C33	1.391 (4)
C3—H3A	0.9800	C32—H32	0.9500
C3—H3B	0.9800	C33—C34	1.371 (5)
C3—H3C	0.9800	C33—H33	0.9500
C4—C5	1.514 (3)	C34—C35	1.381 (5)
C4—H4A	0.9900	C34—H34	0.9500
C4—H4B	0.9900	C35—C36	1.389 (4)
C5—H5A	0.9800	C35—H35	0.9500
C5—H5B	0.9800	C36—H36	0.9500
C5—H5C	0.9800	C41—C42	1.389 (3)
C11—C12	1.393 (3)	C41—C46	1.393 (3)
C11—C16	1.399 (3)	C42—C43	1.384 (4)
C12—C13	1.399 (3)	C42—H42	0.9500
C12—H12	0.9500	C43—C44	1.374 (4)
C13—C14	1.382 (4)	C43—H43	0.9500
C13—H13	0.9500	C44—C45	1.378 (5)
C14—C15	1.389 (4)	C44—H44	0.9500
C14—H14	0.9500	C45—C46	1.381 (4)
C15—C16	1.390 (3)	C45—H45	0.9500
C15—H15	0.9500	C46—H46	0.9500
C16—H16	0.9500		
			
P1—Cu1—P2	113.37 (2)	C15—C16—H16	119.8
P1—Cu1—S1	106.77 (2)	C11—C16—H16	119.8
P2—Cu1—S1	114.30 (2)	C26—C21—C22	118.6 (2)
P1—Cu1—I1	104.637 (18)	C26—C21—P1	118.01 (18)
P2—Cu1—I1	106.669 (17)	C22—C21—P1	123.33 (18)
S1—Cu1—I1	110.709 (17)	C21—C22—C23	120.1 (2)
C1—S1—Cu1	109.37 (7)	C21—C22—H22	119.9
C21—P1—C11	101.52 (10)	C23—C22—H22	119.9
C21—P1—C27	100.74 (10)	C24—C23—C22	120.6 (2)
C11—P1—C27	102.98 (10)	C24—C23—H23	119.7
C21—P1—Cu1	112.59 (7)	C22—C23—H23	119.7
C11—P1—Cu1	116.01 (8)	C23—C24—C25	119.3 (2)
C27—P1—Cu1	120.36 (7)	C23—C24—H24	120.4
C41—P2—C31	103.26 (10)	C25—C24—H24	120.4
C41—P2—C28	101.76 (10)	C26—C25—C24	120.5 (2)
C31—P2—C28	102.47 (10)	C26—C25—H25	119.8
C41—P2—Cu1	118.97 (7)	C24—C25—H25	119.8
C31—P2—Cu1	117.28 (7)	C25—C26—C21	120.9 (2)
C28—P2—Cu1	110.82 (7)	C25—C26—H26	119.5
C1—N1—C2	125.3 (2)	C21—C26—H26	119.5
C1—N1—H1	117.4	C28^i^—C27—P1	114.89 (15)
C2—N1—H1	117.4	C28^i^—C27—H27A	108.5
C1—N2—C4	124.98 (19)	P1—C27—H27A	108.5
C1—N2—H2	117.5	C28^i^—C27—H27B	108.5
C4—N2—H2	117.5	P1—C27—H27B	108.5
N2—C1—N1	117.4 (2)	H27A—C27—H27B	107.5
N2—C1—S1	120.29 (17)	C27^i^—C28—P2	108.66 (14)
N1—C1—S1	122.29 (17)	C27^i^—C28—H28A	110.0
N1—C2—C3	112.4 (2)	P2—C28—H28A	110.0
N1—C2—H2A	109.1	C27^i^—C28—H28B	110.0
C3—C2—H2A	109.1	P2—C28—H28B	110.0
N1—C2—H2B	109.1	H28A—C28—H28B	108.3
C3—C2—H2B	109.1	C32—C31—C36	118.7 (2)
H2A—C2—H2B	107.9	C32—C31—P2	122.5 (2)
C2—C3—H3A	109.5	C36—C31—P2	118.77 (18)
C2—C3—H3B	109.5	C33—C32—C31	119.8 (3)
H3A—C3—H3B	109.5	C33—C32—H32	120.1
C2—C3—H3C	109.5	C31—C32—H32	120.1
H3A—C3—H3C	109.5	C34—C33—C32	121.2 (3)
H3B—C3—H3C	109.5	C34—C33—H33	119.4
N2—C4—C5	109.97 (19)	C32—C33—H33	119.4
N2—C4—H4A	109.7	C33—C34—C35	119.6 (3)
C5—C4—H4A	109.7	C33—C34—H34	120.2
N2—C4—H4B	109.7	C35—C34—H34	120.2
C5—C4—H4B	109.7	C34—C35—C36	120.0 (3)
H4A—C4—H4B	108.2	C34—C35—H35	120.0
C4—C5—H5A	109.5	C36—C35—H35	120.0
C4—C5—H5B	109.5	C35—C36—C31	120.8 (3)
H5A—C5—H5B	109.5	C35—C36—H36	119.6
C4—C5—H5C	109.5	C31—C36—H36	119.6
H5A—C5—H5C	109.5	C42—C41—C46	117.8 (2)
H5B—C5—H5C	109.5	C42—C41—P2	118.50 (18)
C12—C11—C16	119.4 (2)	C46—C41—P2	123.68 (18)
C12—C11—P1	119.57 (17)	C43—C42—C41	121.2 (2)
C16—C11—P1	121.03 (17)	C43—C42—H42	119.4
C11—C12—C13	119.8 (2)	C41—C42—H42	119.4
C11—C12—H12	120.1	C44—C43—C42	120.3 (3)
C13—C12—H12	120.1	C44—C43—H43	119.8
C14—C13—C12	120.5 (2)	C42—C43—H43	119.8
C14—C13—H13	119.7	C43—C44—C45	119.2 (3)
C12—C13—H13	119.7	C43—C44—H44	120.4
C13—C14—C15	119.9 (2)	C45—C44—H44	120.4
C13—C14—H14	120.0	C44—C45—C46	120.8 (3)
C15—C14—H14	120.0	C44—C45—H45	119.6
C14—C15—C16	120.0 (2)	C46—C45—H45	119.6
C14—C15—H15	120.0	C45—C46—C41	120.7 (3)
C16—C15—H15	120.0	C45—C46—H46	119.6
C15—C16—C11	120.3 (2)	C41—C46—H46	119.6
			
C4—N2—C1—N1	−7.2 (3)	P1—C21—C26—C25	177.6 (2)
C4—N2—C1—S1	173.37 (18)	C21—P1—C27—C28^i^	−165.70 (16)
C2—N1—C1—N2	174.1 (2)	C11—P1—C27—C28^i^	−61.09 (18)
C2—N1—C1—S1	−6.5 (3)	Cu1—P1—C27—C28^i^	69.95 (17)
Cu1—S1—C1—N2	−33.6 (2)	C41—P2—C28—C27^i^	66.78 (16)
Cu1—S1—C1—N1	146.99 (17)	C31—P2—C28—C27^i^	173.40 (15)
C1—N1—C2—C3	−90.6 (3)	Cu1—P2—C28—C27^i^	−60.70 (15)
C1—N2—C4—C5	−176.4 (2)	C41—P2—C31—C32	−20.2 (2)
C21—P1—C11—C12	−123.32 (18)	C28—P2—C31—C32	−125.7 (2)
C27—P1—C11—C12	132.67 (18)	Cu1—P2—C31—C32	112.8 (2)
Cu1—P1—C11—C12	−0.9 (2)	C41—P2—C31—C36	161.93 (18)
C21—P1—C11—C16	54.8 (2)	C28—P2—C31—C36	56.5 (2)
C27—P1—C11—C16	−49.2 (2)	Cu1—P2—C31—C36	−65.11 (19)
Cu1—P1—C11—C16	177.20 (16)	C36—C31—C32—C33	−1.1 (4)
C16—C11—C12—C13	−0.7 (3)	P2—C31—C32—C33	−178.9 (3)
P1—C11—C12—C13	177.45 (17)	C31—C32—C33—C34	−0.4 (5)
C11—C12—C13—C14	−0.1 (3)	C32—C33—C34—C35	0.8 (5)
C12—C13—C14—C15	0.8 (4)	C33—C34—C35—C36	0.3 (4)
C13—C14—C15—C16	−0.7 (4)	C34—C35—C36—C31	−1.9 (4)
C14—C15—C16—C11	−0.1 (4)	C32—C31—C36—C35	2.2 (4)
C12—C11—C16—C15	0.8 (3)	P2—C31—C36—C35	−179.84 (19)
P1—C11—C16—C15	−177.35 (18)	C31—P2—C41—C42	114.9 (2)
C11—P1—C21—C26	56.7 (2)	C28—P2—C41—C42	−139.1 (2)
C27—P1—C21—C26	162.44 (19)	Cu1—P2—C41—C42	−17.1 (2)
Cu1—P1—C21—C26	−68.06 (19)	C31—P2—C41—C46	−67.9 (2)
C11—P1—C21—C22	−127.0 (2)	C28—P2—C41—C46	38.1 (3)
C27—P1—C21—C22	−21.2 (2)	Cu1—P2—C41—C46	160.1 (2)
Cu1—P1—C21—C22	108.27 (19)	C46—C41—C42—C43	−0.4 (4)
C26—C21—C22—C23	0.3 (4)	P2—C41—C42—C43	177.0 (2)
P1—C21—C22—C23	−176.0 (2)	C41—C42—C43—C44	−0.7 (5)
C21—C22—C23—C24	−1.5 (4)	C42—C43—C44—C45	1.1 (5)
C22—C23—C24—C25	1.2 (4)	C43—C44—C45—C46	−0.4 (6)
C23—C24—C25—C26	0.2 (4)	C44—C45—C46—C41	−0.7 (6)
C24—C25—C26—C21	−1.3 (4)	C42—C41—C46—C45	1.1 (5)
C22—C21—C26—C25	1.1 (4)	P2—C41—C46—C45	−176.2 (3)

Symmetry code: (i) −*x*+1, −*y*+1, −*z*.

### Hydrogen-bond geometry (Å, º)

**Table:**

*D*—H···*A*	*D*—H	H···*A*	*D*···*A*	*D*—H···*A*
N1—H1···I1^ii^	0.88	2.80	3.622 (2)	156
N2—H2···I1	0.88	2.70	3.5517 (19)	162

Symmetry code: (ii) −*x*+3/2, *y*+1/2, −*z*+1/2.
