# Supplementary figures and images for: Crystal structure of bis­[μ-1,2-bis­(di­phenyl­phosphan­yl)ethane-κ2 P:P′]bis[(N,N′-di­ethyl­thio­urea-κS)iodidocopper(I)]
Source: Acta Crystallogr E Crystallogr Commun. 2015 Aug 6;71(Pt 9):m154–5. doi: 10.1107/S2056989015014176 (PMC4555430; doi:10.1107/S2056989015014176)

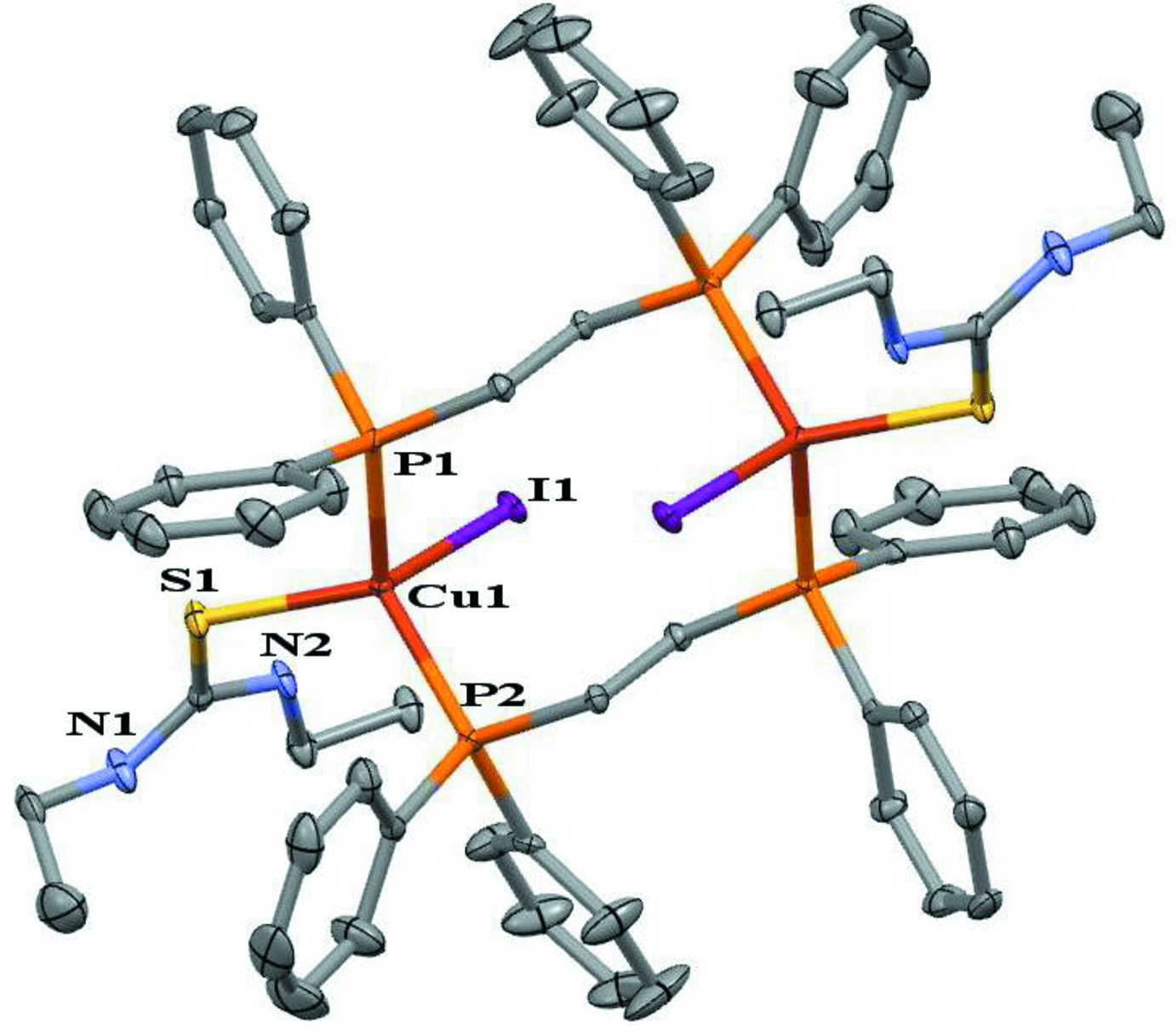

Supplement: Supplementary file 3 [file e-71-0m154-fig1.tif]

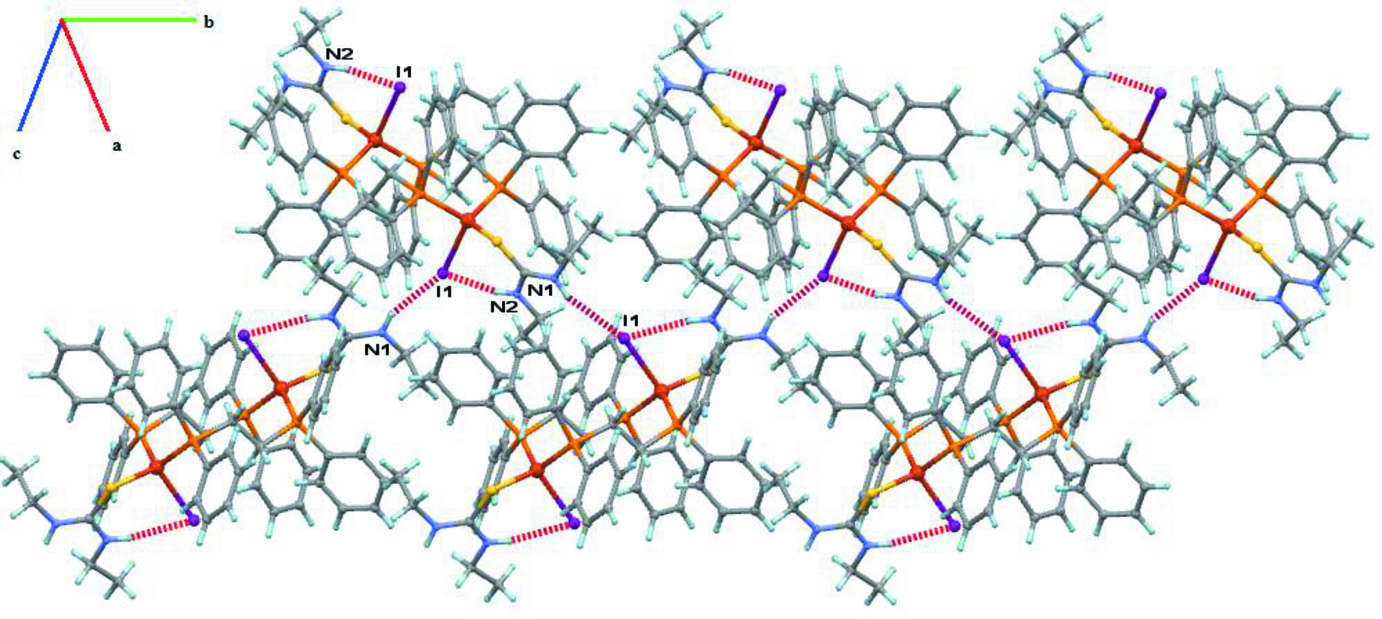

Supplement: Supplementary file 4 [file e-71-0m154-fig2.tif]
